# Equal Prevalence of Genotypes ON1 and BA of Human Orthopneumovirus in Riyadh, Saudi Arabia, in 2022

**DOI:** 10.3390/cimb47100826

**Published:** 2025-10-08

**Authors:** Anwar Ahmed, Abdulkarim Alhetheel, Fahad N. Almajhdi, Shama Parveen, Muslim M. AlSaadi, Khalid F. Al-Mobaireek

**Affiliations:** 1 Centre of Excellence in Biotechnology Research, College of Applied Medical Sciences, King Saud University, Riyadh 11413, Saudi Arabia; 2 Department of Pathology & Laboratory Medicine, College of Medicine, King Khalid University Hospital, King Saud University, Riyadh 12372, Saudi Arabia; 3 Department of Botany & Microbiology, College of Science, King Saud University, Riyadh 11451, Saudi Arabia; 4 Centre for Interdisciplinary Research in Basic Sciences, Jamia Millia Islamia, New Delhi 110025, India; 5 Department of Pediatrics, College of Medicine, King Khalid University Hospital, King Saud University, Riyadh 12372, Saudi Arabia

**Keywords:** Human orthopneumovirus, respiratory syncytial virus, ON1 genotype, BA genotype, phylogenetic analysis, mutational analysis, glycosylation sites, Saudi Arabia

## Abstract

Human orthopneumovirus (HOPV) is a major cause of acute respiratory tract infection (ARI) in children around the world. The present study was conceptualized to detect and characterize human orthopneumovirus in 640 NPAs collected from symptomatic ARI pediatric patients younger than 2 years of age. The samples were collected from a hospital in Riyadh, Saudi Arabia, during winter 2022. Orthopneumovirus was detected in 98 (15.31%) of the 640 NPAs. No significant difference in the prevalence of HOPV-A (49%) and HOPV-B (51%) was observed during the study period as they circulated at similar frequencies. The HOPV-A strains (33) and HOPV-B strains (47) clustered into ON1 and BA genotype, respectively. The ON1 genotypes were further categorized into the subgenotype GA-2.3 and three different lineages, GA-2.3.5, GA-2.3.6a, and GA-2.3.6b, whereas the BA genotypes were categorized into the GB-5.0 subgenotype, entirely belonging to the GB-5.0.5a lineage. This is the first report to characterize orthopneumovirus strains from Saudi Arabia using a recently reported method. Several mutations, a few N-/O-glycosylation sites, and some purifying selections were observed in both the ON1 and BA genotypes. The present study demonstrates the equal prevalence of the ON1 and BA genotypes, in contrast to earlier reports on HOPV-A prevalence in the region. Understanding the change in the genotype distribution of HOPV requires the uninterrupted surveillance and genetic characterization of HOPV in circulating respiratory infections. These findings may contribute to a better understanding of HOPV evolution and the dynamics of its distribution at the local and global levels, resulting in improved understanding of epidemics.

## 1. Introduction

Human orthopneumovirus (HOPV), also known as Respiratory Syncytial Virus (RSV), is a major cause of acute respiratory tract infections (ARIs) and causes approximately 33 million infections (ranging between 25.4 and 44.6 million) and 3.6 million hospital admissions annually in children below 5 years of age across the globe, leading to approximately 100,000 (84,500–125,200) deaths [1]. It is a leading cause of hospitalization of infants and the second most prevalent cause of death from lower respiratory infections, with 54% of deaths being attributed to human orthopneumovirus in children younger than 5 years of age [2]. Higher mortality rates due to human orthopneumovirus infections are also seen in people aged over 65 years and in adults with underlying medical complications. Human orthopneumovirus has a linear single-stranded RNA genome approximately 15 kb in length and is an enveloped virus [3,4]. The virus genome encodes 11 proteins. The attachment protein (G protein) of orthopneumovirus attaches the virion to the host cell. The antigenic reactivity of the G gene of orthopneumovirus is used for its classification into two subgroups, hRSV-A and hRSV-B (also known as HOPV-A and HOPV-B, respectively), which are further classified into genotypes based on genetic variation within the gene [5]. The G protein is located on the surface of the virion and, therefore, as an antigen, has a neutralizing capacity and is thus a vaccine candidate [6]. The G protein has an ectodomain with two hypervariable regions, I and II. Hypervariable region II is a hotspot for mutations and has therefore been used for human orthopneumovirus genotyping in epidemiological studies [7,8,9]. Genotype classifications of orthopneumovirus based on the entire G gene sequence have also been reported [10,11].

Recent studies have focused more on the ectodomain region of the G protein gene for human orthopneumovirus classification as the ectodomain sequence-based classification shows similar topologies to full-length G gene sequences or full-genome sequences of human orthopneumovirus [12]. Hypervariable region II in the ectodomain of the G protein had shown a major insertion of 60 bp in the BA genotype of HOPV-B in 1999 from Argentina [13], followed by a 72 bp duplication in the same region of the ON1 genotype of HOPV-A from Canada in 2011 [14]. These duplication regions are associated with mutations that lead to the development of variants or lineages within these genotypes [12,15,16]. These mutations are acquired by new genotypes to evade host immune systems and thus have a better chance of survival [17]. In due course, these genotypes spread to other geographical areas due to immunologically naive populations and become endemic [18].

Classification based on G protein gene sequences has provided information on the transmission dynamics and co-circulation of different genotypes of both subgroups of orthopneumovirus during epidemics [5,9,19]. This classification has also provided knowledge on the displacement of dominant genotypes over successive epidemic seasons [8,20,21]. A few studies have reported the potential of a particular orthopneumovirus genotype to predominate for a particular period of time within a community [11,16,22].

Studies on HOPV classification have currently defined 13 HOPV genotypes among the subgroup A strains (GA1-7, SAA1, NA1-4, and ON1-2) and 20 genotypes among the subgroup B strains (GB1-4, SAB1-4, URU1-2, and B A1-10) [12]. These obtained phylogenies differ according to the different phylogenetic methods used. Variable definitions of significant bootstrap values, different lengths of sequence fragments used for analysis, average genetic distance (p-distance), and arbitrarily selected cut-off values or similarity values result in a lack of consensus for genotype allocation. Recently, Goya and colleagues [12] proposed a unified molecular method of HOPV surveillance based on both phylogenetic analyses and average p-distances to analyze the HOPV strains. The G-ectodomain region was chosen as the minimum region suitable for HOPV genotyping since the topologies obtained from whole genome sequences and G-ectodomain sequences were almost similar. They reduced the number of genotypes of HOPV-A from 13 to 3 (GA1–GA3) and from 20 to 7 for HOPV-B (GB1–GB7). These genotypes were further classified to two additional levels as subgenotypes and lineages.

A few studies have been conducted on molecular characterization of HOPV in Saudi Arabia [7,8,9,23]. These studies were carried out using HOPV classification methods reported earlier than the unified molecular method of HOPV surveillance reported by Goya and colleagues [12]. The present study was therefore designed to evaluate and characterize the HOPV strains circulating in Riyadh, Saudi Arabia, during the winter of 2022 using the newly reported classification method [12] and compare them to the HOPV strains reported earlier in the region. Human orthopneumovirus clustered into the ON1 and BA genotypes with 72 bp and 60 bp duplication in the G protein gene, respectively. The ON1 genotypes were further categorized into subgenotype GA-2.3 and three different lineages, GA-2.3.5, GA-2.3.6a and GA-2.3.6b, whereas the HOPV-B BA genotype was categorized into the GB-5.0 subgenotype, entirely belonging to the GB-5.0.5a lineage. This is the first report on characterization of orthopneumovirus strains from Saudi Arabia using the recently reported method.

## 2. Materials and Methods

### 2.1. Ethical Approval

The study was approved (E-19-4498) by the Institutional Review Board of the College of Medicine at King Saud University (KKUH), Riyadh, Saudi Arabia.

### 2.2. Sample Collection

Six hundred and forty samples (nasopharyngeal aspirates) were collected from the Pediatrics Department of the College of Medicine at King Khalid University Hospital (KKUH), King Saud University Riyadh, Saudi Arabia. The protocol followed was that suggested by Al-Haseenah and colleagues in 2020 [9]. Briefly, children younger than 2 years of age admitted to the ward or attending the emergency department with ARI symptoms in November and December 2022 were enrolled for the study. NPAs were collected in 3 mL UTM Copan viral collection tubes (UTM^®^ Copan, Brescia, Italy) and transported to the Centre of Excellence in Biotechnology Research Lab, where the samples were processed and stored at −80 °C until RNA was extracted.

### 2.3. RNA Extraction, cDNA Synthesis, and Orthopneumovirus Detection

RNA was extracted using the RNeasy Mini Kit (Qiagen, CA, USA) from 500 μL of sample as described earlier [7]). Briefly, the samples were lysed after thawing and vortexed in 1 volume of lysis buffer (RLT) containing 1% β-mercaptoethanol. RNA was precipitated by the addition of a 1:1 volume of 70% ethanol and allowed to bind to the silica membrane on the RNeasy Mini spin column by passing the lysate through it. The column was washed with 700 μL of (RW1) buffer followed by 500 μL of (RPE) buffer. Elution was achieved by adding 50 μL of RNase-free water. The spin column was centrifuged for 30 s at 8000× *g* for all steps except the elution step where the centrifugation time was 1 min. RNA was quantitated and transcribed to cDNA with a High-Capacity cDNA Reverse Transcription Kit (Applied Biosystems, Carlsbad, CA, USA) using random primers following the manufacturer’s instructions. Briefly, each 20 μL reaction contained 2.0 µL of 10 × reverse transcription buffer, 0.8 µL of 25 × dNTP mix, 2.0 µL of 10 × reverse transcription random primers, 1.0 µL of multiScribe reverse transcriptase, 10 µL of RNA preparation, and 4.2 µL of nuclease-free water. The reaction mixtures were incubated at 25 °C for 10 min, 37 °C for 120 min, and 85 °C for 5 min to convert RNA into cDNA, and were stored at −20 °C until used.

cDNA (2 μL) was used for the amplification of the L gene of orthopneumovirus with TaqMan^®^ technology using the AgPath-ID™ One-Step RT-PCR kit (Appied Biosystems, USA) for a real-time PCR run in a 7500 real-time PCR system (Appied Biosystems, USA). The cycling conditions for the reaction were 1 cycle at 95 °C for 3 min * (DNA polymerase activation), 40 PCR cycles of 95 °C for 15 s, and 60 °C for 1 min. The forward oligo qRSV-ABLF (5′-GTGGAACTTCATCCTGACATAAGATATATT-3′), the reverse oligo qRSV-ABLR (5′-GTTGCATCTGTAGCAGGAATGGT-3′), and the probe qRSV-ABLPRB (5′-FAMATTGCAATGATCATAGTTTACCT-MBG-3′) were used as suggested [23]. With proper controls, the presence of orthopneumovirus genetic material was confirmed by increased fluorescence signals.

### 2.4. Amplification and Sequencing of G Gene

Orthopneumovirus G protein gene ectodomain amplification was performed using the forward oligo BG10F-GCAATGATAATCTCAACCTC and reverse oligo F1-CAACTCCATTGTTATTTGCC with the suggested parameters [24]. Briefly, thermocycling was performed for initial denaturation at 95 °C for 2 min, followed by 40 cycles of 95 °C for 30 s, 55 °C for 30 s, and 72 °C for 1 min, followed by a final extension at 72 °C for 5 min. The amplicons were visualized through agarose gel electrophoresis using 1.5% agarose gel in a UVP GelDoc Imaging system (UVP, Cambridge, UK). Specific amplicons were cut from the gel and DNA was extracted using a QIAquick Gel Extraction Kit (QIAGEN, Valencia, CA, USA). The extracted DNA was quantified using a NanoDrop 1000 Spectrophotometer (Thermo Fisher science, Waltham, MA, USA). The amplicons were sequenced commercially (Macrogen, Seoul, Republic of Korea) using the Sanger sequencing method in both forward and reverse directions with the same forward (BG10F) and reverse (F1) oligos as used for G protein gene ectodomain amplification. The average read length of the template was 1000 bases. Sequences were BLAST-searched for orthopneumovirus A and B genotypes at https://blast.ncbi.nlm.nih.gov/Blast.cgi (accessed on 7 July 2025) and edited manually and aligned using MEGA12 and BioEdit software version 7.2.6.1. The sequences were trimmed at both terminals to obtain the ectodomain region of the HOPV-A and HOPV-B genotypes.

### 2.5. Phylogenetic Analysis

The ectodomain sequences of orthopneumovirus genotypes A and B and the sequences downloaded from GenBank (Tables S1 and S2) were aligned using the CLUSTAL W algorithm in BioEdit software version 7.2.6.1. The sequences were edited using the same BioEdit software. The Maximum Likelihood method in MEGA12 was used for the phylogenetic tree construction with a robustness of 1000 bootstrapping replicas. Genetic distances were calculated using the Kimura 2 method of nucleotide substitution [25]. The JN257693 and DQ227364 prototype strains were used for the HOPVA-ON1 and HOPVB-BA genotypes, respectively.

### 2.6. Amino Acid Sequence and Selection Pressure Analysis

The amino acid sequence analysis for the HOPV-A and HOPV-B G protein ectodomains was carried out with their respective prototype strains using BioEdit software as described earlier [9]. Selection pressure analysis of the ectodomain was carried out using the Datamonkey Adaptive Evolution Server (http://www.datamonkey.org/) [26]. The mixed effects model of evolution (MEME) and fast unconstrained Bayesian approximation for inferring selection (FUBAR) methods were used for analysis. A consensus was used to calculate synonymous (dS) and non-synonymous (dN) rates per codon of the amino acid alignment. The positive selected sites (dN/dS > 1) and negative selected sites (dN/dS < 1) were determined using a *p*-value of 0.1 (MEME) or a posterior probability of 0.9 (FUBAR).

### 2.7. Entropy Analysis

Variations in amino acids at the ectodomain of the G protein gene were analyzed by Shanon entropy plot in BioEdit software as described earlier [8]. The analysis was carried out in all 33 HOPV-A and 47 HOPV-B sequences. The entropy values ranged between 0 and 1.8, with a threshold value of 0.2. Entropy values <0.2 and >0.2 show conserved and variable amino acid sites, respectively [27].

### 2.8. N- and O-Linked Glycosylation Sites

Glycosylation at potential O- and N-linked sites of the G gene ectodomain was predicted with the prototype strains JN257693 and DQ227364 for the HOPV-A and HOPV-B genotypes, respectively, using the NetNGlyc1.0 (http://www.cbs.dtu.dk/services/NetNGlyc) and NetOGlyc3.1 (http://www.cbs.dtu.dk/services/NetOGlyc) servers [28]. Ser and Thr provide locations for O-glycosylation while N-glycosylation occurs on Asn in the sequence motif Asn-Xaa-Ser/Thr (where Xaa is any amino acid except proline). The sequence sites with a threshold potential greater than 0.5 were marked as glycosylated sites.

### 2.9. Statistical Analysis

A Chi-square goodness-of-fit test was used to determine whether the prevalence of HOPV-A and HOPV-B among RSV-positive samples differed statistically significantly. According to the null hypothesis, the two genotypes were distributed equally (expected proportion 50:50). The following formula was used to compare observed frequencies to expected counts:

$$\chi^{2}=\sum \frac{{\left(O_{i}-E_{i}\right)}^{2}}{E_{i}}$$

where *Oᵢ* represents the observed frequency and *Eᵢ* represents the expected frequency. Statistical significance was set at *p* < 0.05.

## 3. Results

### 3.1. Patient Characteristics

A total of 640 clinical specimens were collected from children with ARI symptoms in November and December 2022. The mean age of the patients was 7.5 months. The 1–6 months age group showed a maximum prevalence of 45.3%, while prevalence in the patients in the 6–12 months and 13–24 months age groups were 32%, and 22.7%, respectively. Considering the demographic distribution, more male patients (59.8%) were reported than female patients (40.2%) (Table 1).

### 3.2. Orthopneumovirus Prevalence, DNA Sequencing, and GenBank Accession Numbers

Orthopneumovirus was detected in 98 (15.31%) of the 640 samples by real-time PCR targeting the L gene using TaqMan probe technology. DNA sequence analysis of the orthopneumovirus G protein gene ectodomain categorized them into group A HOPV in 48 (49%) and B HOPV in 50 (51%) of the samples. Eighteen amplicons (15 HOPV-A and 3 HOPB-B) could not be sequenced properly and were omitted from the analysis. All the sequences of group A HOPV (33) belong to the ON1 genotype with a 72 bp duplication in the G gene, while all of the group B HOPV (47) isolates belong to the BA genotype with a 60 bp duplication in the G gene. The calculated expected amplicon size of the HOPVA-ON1 genotype ectodomain fragments includes 828 bp of the 3′G gene, while the BA genotype ectodomain fragments include a 795 bp 3′G gene region. Complete ectodomain regions of both genotypes were used for analysis and alignment. All the sequences were deposited to GenBank (https://www.ncbi.nlm.nih.gov/genbank/) with accession numbers PQ798904 to PQ798936 for the HOPV-A G protein gene ectodomain and PQ798831 to PQ798877 for the HOPV-B G protein gene ectodomain region (Table S3).

### 3.3. Phylogenetic Analysis

#### 3.3.1. HOPV-A Strains

**Figure 1:**
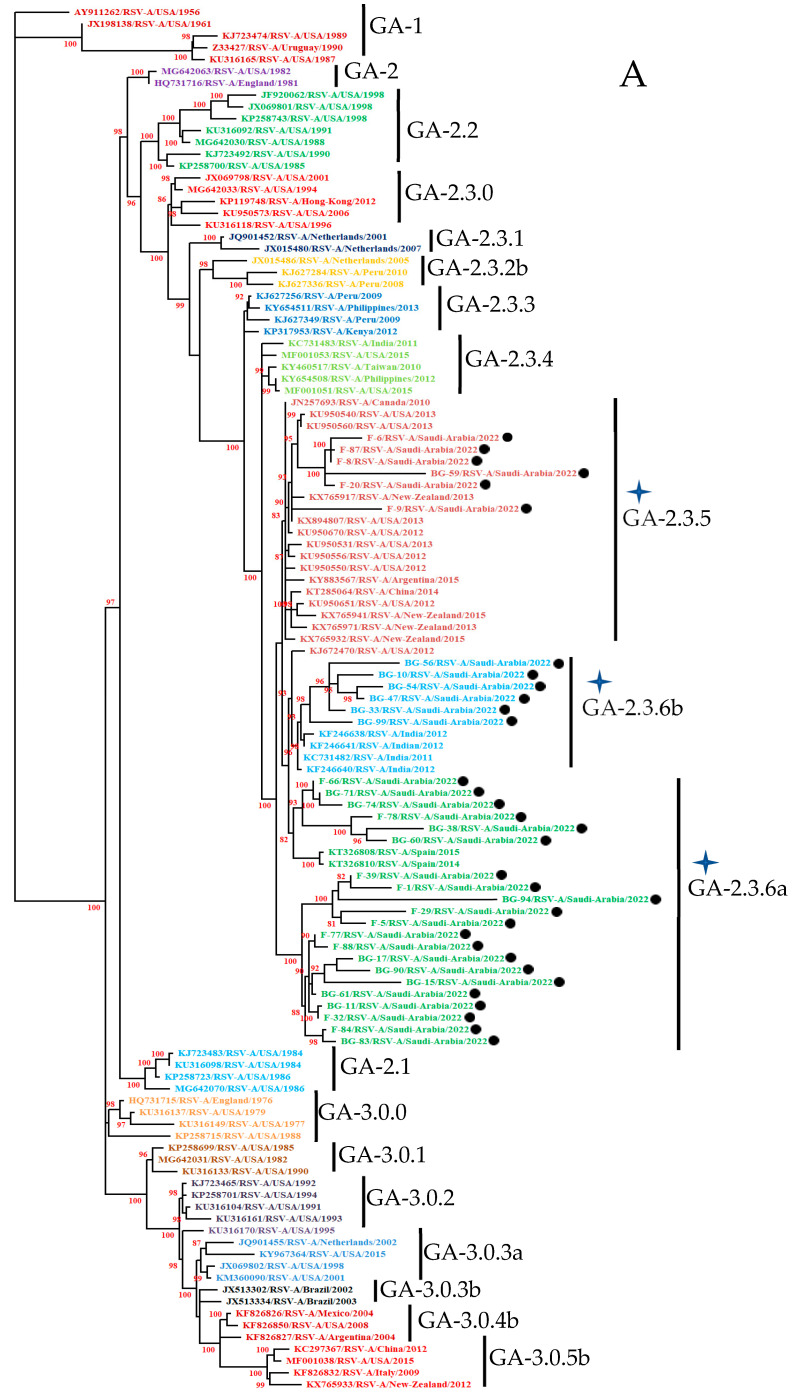

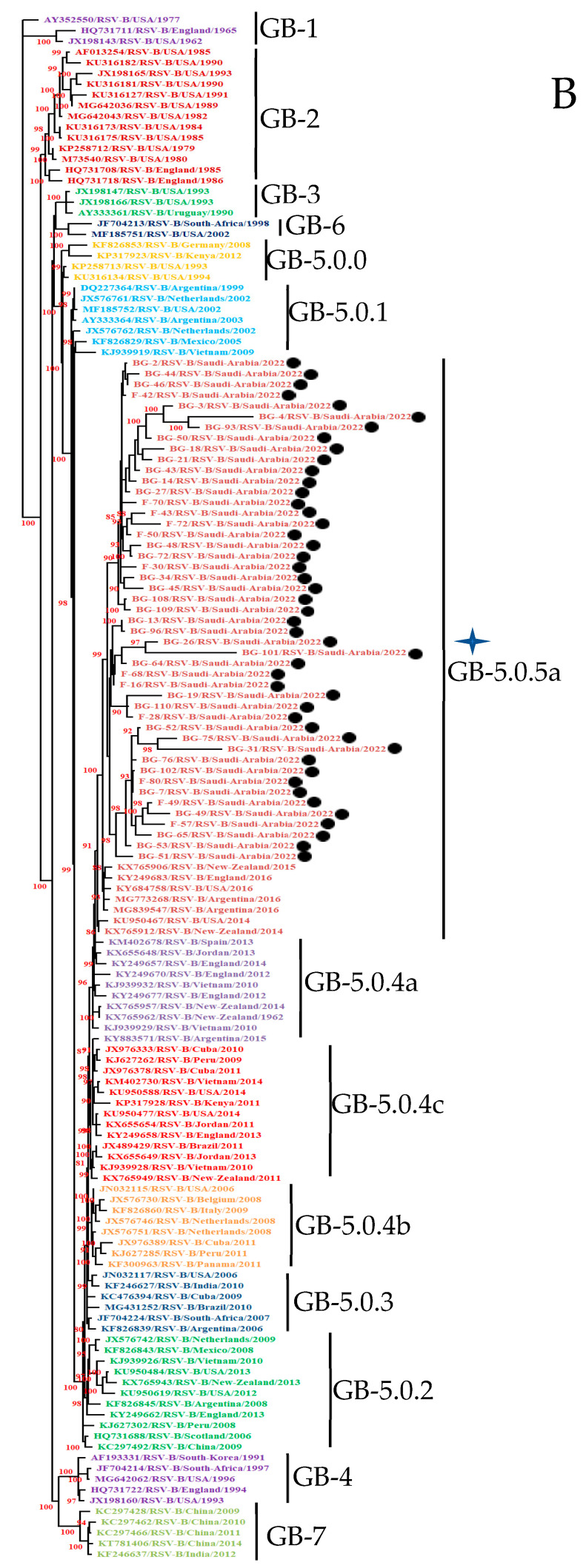
Phylogenetic trees from the nucleotide sequences of the ectodomain of the G protein gene of group A orthopneumovirus (**A**) and group B orthopneumovirus (**B**). The tree was constructed by the Maximum Likelihood method using Kimura-2 parameters with 1000 bootstrapping replicas. Stars indicate study strains with genotype, subgenotype, and lineage. Close circle (●) indicate study sequences from Riyadh, Saudi Arabia, during November and December, 2022.

A total of one hundred and seventeen nucleotide sequences (thirty-three study sequences and eighty-four reported sequences at GenBank) of the ectodomain of the G protein gene of HOPV-A were used in this study. A 768 bp ectodomain region of the G protein gene in the ON-1 prototype strain of HOPV-A was used for alignment. Phylogenetic analysis of all (33) HOPV-A G gene ectodomain regions categorized them into the HOPVA-ON1 genotype (Figure 1A). The ON1 genotype was further categorized into subgenotype GA-2.3 with three different lineages, GA-2.3.5, GA-2.3.6a, and GA-2.3.6b, as mentioned earlier [12]. The prototype strain used in this study was JN257693 for the HOPVA ON-1 genotype. The nucleotide distances between the prototype and study sequences and among all study sequences were 1.45–5.97% and 0.13–11.61%, respectively. The amino acid distances between the prototype and study sequences and among all study sequences were 2.78–16.13% and 0–23.79%, respectively (Table S4).

#### 3.3.2. HOPV-B Strains

A total of one hundred and forty-four nucleotide sequences (forty-seven study sequences and ninety-seven sequences reported at GenBank) of the ectodomain of the G protein gene of HOPV-B were used. A 735 bp ectodomain region of the G protein gene in the BA prototype strain of HOPV was used for alignment. Sequence determination of ectodomain of the G protein gene and phylogenetic analysis categorized the HOPV strains into the HOPV-B BA genotype (47). The BA genotype was further categorized into subgenotype GB-5.0 and a GB-5.0.5a lineage, as mentioned earlier [12]. The nucleotide and protein distances were calculated as shown earlier. The prototype strain used in this study was DQ227364 for the HOPVB BA genotype. All 47 study sequences clustered in the BA genotype (Figure 1B). The nucleotide distances between the prototype and study sequences and among all study sequences were 3.94–16.29% and 0–20.46%, respectively. The amino acid distances between the prototype and study sequences and among all study sequences were 6.34–28.22% and 0–36.13%, respectively (Table S5).

### 3.4. Mutational Analysis

The sequences of the study HOPV-A and HOPV-B G protein genes were compared with their respective prototype strains.

#### 3.4.1. Deduced Amino Acid Sequences and Mutational Analysis of HOPVA-ON1 Genotypes

**Figure 2:**
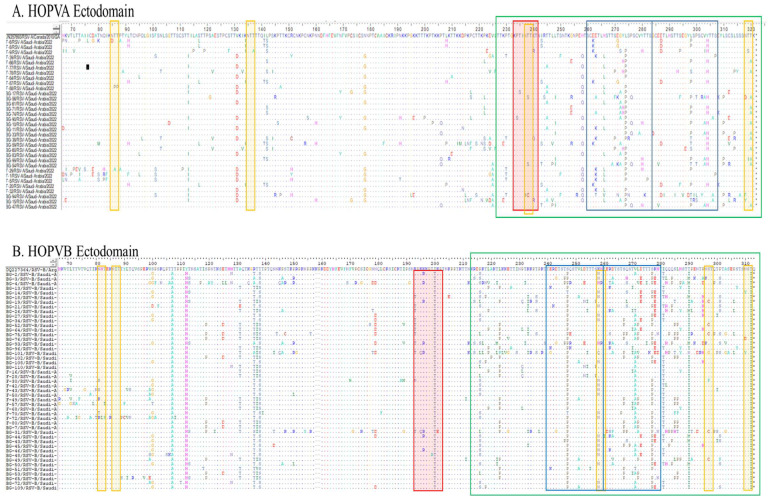
Deduced amino acid sequence alignment and mutations of the ectodomain of G protein gene. (**A**) Alignment of Saudi ON1 strains with the prototype strain from Canada (JN257693). The sequence corresponds to 67–321 amino acids of the prototype strain. The second hypervariable region with maximum number of variations within a total stretch of 95 amino acids is shown in green box. Two copies of duplicated 24-amino-acid region in group ON1 strains are indicated by rectangular blue boxes. (**B**) Alignment of Saudi BA strains with the prototype BA strain from Argentina (DQ227364). The sequence corresponds to 67–315 amino acids of the prototype strain. The second hypervariable region with maximum number of variations within a total stretch of 100 amino acids is shown in green box. Two copies of duplicated 20 amino-acid region in group BA strains are indicated by rectangular bblue boxes. “*” represents stop codons, yellow shade indicate potential N-glycosylation sites (NXT, where X is not proline) red shade represents potential O-glycosylation KPX - - - TTKX motifs.

The amino acid sequences of 33 study sequences were deduced using BioEdit software. The mutations were analyzed by comparison with the prototype Canadian ON-1 strain sequence (JN257693) (Figure 2A). The protein length of all the 33 study sequences of the HOPV-A ON-1 genotype was predicted to be 321 amino acids with corresponding prototype strain (JN257693). The ectodomain region consists of 255 amino acids and variations were observed in 129 sites, with 126 sites being conserved. The second hypervariable region showed the maximum number of variations within a total stretch of 95 amino acids. In an analogous region, a total of 15 mutation sites were observed, while in a duplicated region, a higher number of variable sites were seen (n = 18). No premature termination of the protein or extensions were observed in any of the 33 reported sequences. The E262K and E263K substitutions were observed in an analogous region while the corresponding site at a duplicated region showed an E287D substitution. The H266L substitution was observed in an analogous region while no such substitution was observed in the corresponding duplicated region. The 274 and 298 corresponding sites were highly variable in both analogous and duplicated regions, and the substitution was common at both sites (L to P). The analogous region has a lower frequency of Y280H substitutions compared to the duplicated region (Y304H).

#### 3.4.2. Deduced Amino Acid Sequences and Mutational Analysis of HOPVB-BA Genotypes

Similarly, the amino acid sequences of 47 study sequences were deduced to contain mutations which were analyzed by comparison with the prototype Argentinean BA strain sequence (DQ227364) (Figure 2B). The protein length of all the 47 study sequences of the HOPVB-BA genotype was 312 amino acids, with the exception of two sequences (BG-14/RSV-B/Saudi-Arabia and BG-21/RSV-B/Saudi-Arabia), which have additional amino acids at the C-terminal. The ectodomain is 247 amino acids with variations observed in 157 sites and with 90 sites being conserved. Maximum amino acid variability was seen in the second hypervariable region within a total stretch of 100 amino acids. Thirteen mutation sites were observed in the analogous region (241–260 amino acids) while a higher number of variable sites (17) was seen in the duplicated region (261–179). Extension was observed in two sequences, while the other sequences exhibited no premature protein termination. Eleven of the amino acid sites showed almost complete substitution in the ectodomain region. These sites include T107A, Y112H, R136T, T138S, I200T, S247P, V271A, S277P, I281T, T290IM, and T312I. The analogous region has a lower frequency of mutations compared to the duplicated region.

### 3.5. Entropy Analysis

#### 3.5.1. Entropy Analysis of HOPVA-ON1 Genotypes

**Figure 3:**
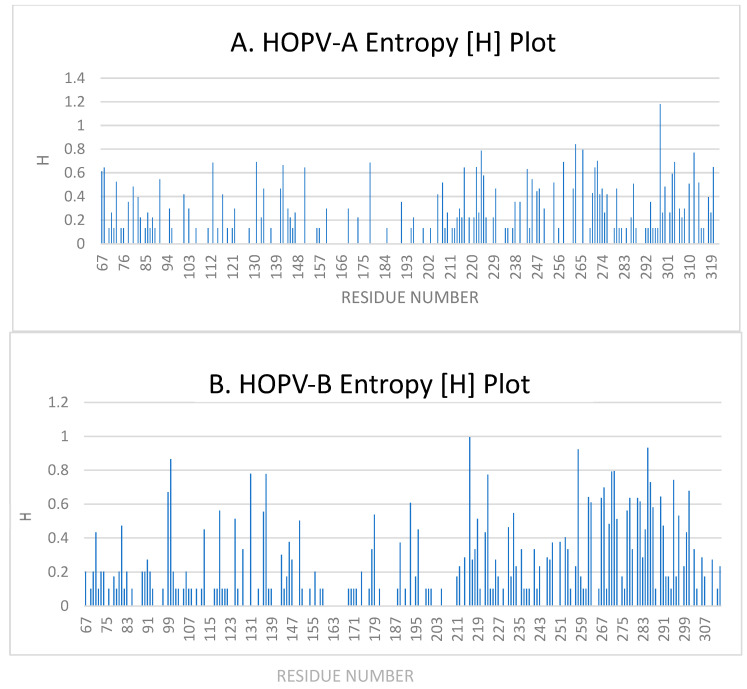
Shannon entropy plots of the ectodomain of the G protein of the HOPVA ON1 genotype (**A**) and the HOPVB BA genotype (**B**).

Shannon entropy analysis was carried out in the ectodomain region of the G protein gene as suggested above. The entropy range is 0–1.8, with a threshold value of 0.2. The Shannon entropy analysis of the ectodomain of the G protein was carried out in all 33 study sequences (Figure 3A). This analysis reveals 129 variable sites and 127 conserved sites. Forty-three sites have entropy values between 0.13 and 0.2, while fifty-five sites have entropy values between 0.2 and 0.5. Entropy values between 0.5 and 0.7 were observed at 24 sites, while six sites have high entropy values with a maximum entropy value of 1.18 seen at the 298 amino acid position where the substitution is L298P/S/T.

#### 3.5.2. Entropy Analysis of HOPVB-BA Genotypes

Similarly, Shannon entropy analysis was carried out for the HOPVB-BA genotypes G protein gene ectodomain region. The entropy range is 0–0.99, with a threshold value of 0.2. Figure 3B shows the Shannon entropy analysis of the ectodomain of the G protein for all 47 study sequences. Variable sites were seen at 157 positions, while conserved sites were seen at 90 amino acid positions. Entropy values between 0.101 and 0.2 were observed at 72 sites, while 52 sites had entropy values between 0.2 and 0.5. Higher entropy values, between 0.5 and 0.7, were observed at 21 sites, while eleven sites had the highest entropy values with a maximum entropy value of 0.99 seen at the 216 amino acid position where the substitution is P216S/L.

### 3.6. O- and N-Linked Glycosylation Sites in HOPV-A and HOPV-B Genotypes

#### 3.6.1. O- and N-Linked Glycosylation Sites in HOPVA-ON1 Genotypes

The potential O- and N-linked glycosylation sites of HOPV-A were predicted with the prototype strain (JN257693) as shown earlier. A total of 33 study sequences were used in this analysis. N-glycosylation occurs on asparagine in the sequence motif Asn-Xaa-Ser/Thr (where Xaa is any amino acid except proline). Among the 33 study sequences, each sequence shows approximately four potential N-glycosylation sites, which is similar to the prototype sequence (Figure 2A). The most likely N-glycosylation sites are amino acid positions 85, 135, 237, and 318. Of the total 132 (33 samples × 4 sites) potential N-glycosylation sites, glycosylation was lost at 22 sites in different samples due to mutation incorporation. O-linked glycosylation is based on the amino acids, serine and threonine. Only a single site at 238 showed potential for O-glycosylation. Some sites lost the potential for O-glycosylation because of the substitution of Pro with Ser at the 234 amino acid position, the substitution of Thr with Cys at the 238 amino acid position, and the substitution of Lys with Arg/Glu at the 240 amino acid position.

#### 3.6.2. O- and N-Linked Glycosylation Sites in HOPV-BA Genotypes

Similarly, the potential O- and N-linked glycosylation sites of HOPV-B were predicted in the prototype strain (DQ227364) and 47 study sequences. Each study sequence shows approximately 4–5 potential N-glycosylation sites (Figure 2B), with the most likely N-glycosylation occurring at amino acid positions 83, 88, 260, 298, and 312. The N-glycosylation site at position 88 is present in all the samples and the prototype strain, and the 312 N-glycosylation site is not present in any of the study sequences but is present in the prototype strain. A loss of glycosylation was seen at 11 out of a total 188 potential N-glycosylation sites in different samples due to mutation incorporation. The O-linked glycosylation site was not observed in the prototype strain but was present in the study sequences at the 199 amino acid position. Some sites lost the potential for O-glycosylation because of the substitution of K193T/N, T199A, and K201E in the study sequences.

### 3.7. Selection Pressure Analysis

The selection pressure was analyzed using Datamonkey Adaptive Evolution Server (www.datamonkey.org). The mixed effects model of evolution (MEME) and fast unconstrained Bayesian approximation for inferring selection (FUBAR) methods were used for this analysis.

#### 3.7.1. ON1 Genotype

Analysis of selection pressure in the HOPVA-ON1 genotypes using the MEME and FUBAR methods showed thirteen purifying selection sites and eight diversifying selection sites. Sites 73, 217, 238, 272, and 298 show diversifying selection by both methods, while sites 72, 82, 178, 223, and 293 were positive using the MEME method only. Sites 80, 81, and 312 were positively selected by the FUBAR method only (Table S6).

#### 3.7.2. BA Genotype

Selection pressure analysis of the HOPVB-BA genotypes showed twenty-five purifying and three diversifying selection sites. Sites 74, 231, and 293 showed diversifying selection by both the MEME and FUBAR methods. Sites 71, 73, 79, 81, 83, 89, 92, 99, 110, 113, 126, 128, 150, 168, 177, 222, 234, 244, 248, 266, and 298 were positive using the MEME method of selection only, while the majority of sites did not show positive selection using the FUBAR method (Table S7).

### 3.8. Statistical Analysis

Ninety eight of the 650 samples were found to be RSV-positive. Of these, 50 (51%) were found to have HOPV-B and 48 (49%) to have HOPV-A. Forty nine was the expected frequency for each genotype under the null hypothesis of equal distribution. The value of Chi-square is:

$$\chi^{2}=\frac{{\left(48-49\right)}^{2}}{49}+\frac{{\left(50-49\right)}^{2}}{49}$$

$$\chi^{2}=\frac{1}{49}+\frac{1}{49}=\frac{2}{49}\approx 0.04$$

There was not a significant deviation from the expected equal distribution, as indicated by the corresponding *p*-value of 0.84 with 1 degree of freedom (df). This suggests that the prevalence of HOPV-A and HOPV-B was almost the same.

## 4. Discussion

Human orthopneumovirus is associated with a substantial public health and economic burden due to its high incidence and the limited preventive options available. Infection by the virus generally causes mild disease but severe disease has been reported in children with compromised lung function [29] or with other health complications. Orthopneumovirus is widespread worldwide, including in Saudi Arabia, and mainly affects infants during the winter season. The most severe orthopneumovirus infections have been observed in children younger than 2 years of age, which coincides with data reported globally [9,30,31,32].

The present study was conceptualized to investigate the prevalence of orthopneumovirus in children hospitalized in Riyadh, Saudi Arabia. Molecular characterization of the circulating strains was carried out using phylogenetic, mutational, entropy, and selection pressure analyses. In this study, most of the enrolled patients were in the 0±6 months age group, which is similar to enrolments of this age group in earlier studies [9,31]. Males outnumber females in terms of orthopneumovirus infection, and this gender bias may be due to the fact that more male children were recruited for the study than females, as has been reported in previous studies [7,8,14,31,33,34,35,36]. The patients showed some common clinical symptoms such as fever and nasal discharge. This study was designed to scrutinize orthopneumovirus circulating in Riyadh during the winter of 2022. Real-time PCR detection resulted in the identification of orthopneumovirus in 15.31% (98/640) of the samples. A similar prevalence was reported in Riyadh in an earlier study also using real-time PCR detection [37]. In the Eastern Province of Saudi Arabia, between January 2015 and February 2022, orthopneumovirus was reported in 26.3% (336/1279) of analysed samples [38]. In another study, HOPV was the most common pathogen and caused 54.4% (230/423) of the infections in children in southwest Saudi Arabia [39]. A varying percentage of orthopneumovirus infections has been reported in the Riyadh region [7,8,9,39,40], and the variation may be due to differences in the sensitivities of the methods used for virus detection, sample processing, and sample collection [41].

Due to the varying prevalence of different orthopneumovirus genotypes in the region, regular surveillance is required to provide data on the virus’ evolutionary dynamics. This study describes the molecular characterization of orthopneumovirus strains circulating in the Riyadh region in November and December 2022. Orthopneumovirus subgroups A and B were in circulation during the study period, with HOPV-B being the dominant genotype, accounting for 51% (50) of the patients, as compared to HOPV-A, which was found in 49% (48) of the patients. The analysis showed no statistically significant difference in the prevalence of HOPV-A and HOPV-B during the study period, and that they circulated at similar frequencies. This is in contrast to most previous studies, which had reported on the predominance of HOPV-A in the region [7,9,14,37]. HOPV-A was detected in 59% (23/39) [42] and HOPV-B in 41% (16/39) [43] of the positive samples. A study conducted by Ahmed and colleagues identified HOPV-A in 77% of the hospitalized children while HOPV-B was detected in 23% of patients in Riyadh in 2014 [7]. Another study also showed the predominance of HOPV-A (72%) compared to HOPV-B (24%) in the region in 2016 [9]. A study from Jeddah reported orthopneumovirus A dominance during January to December 2017 [44]. Some studies have reported the predominance of HOPV-B in the region [3,45] and around the world [46,47]. Therefore, no genotype consistency exists across the world in terms of the predominance of particular genotypes in a given area. The equal and variable prevalences emphasise the importance of continuous molecular surveillance in order to identify any future changes in genotype predominance that might have effect on the burden of disease and the development of vaccines.

HOPV in other Arab Gulf States showed a varied prevalence that ranged between 4% and 82% in different studies on hospitalized subjects while the range was smaller (between 6% and 36%) in studies on outpatient subjects [48]. In Kuwait, HOPV was detected in 17% (84/490) of the samples where 64 (76%) isolates were HOPV-A and 20 (24%) isolates were HOPV-B [49]. In another study, HOPV was detected in 13% of children with ARI during 2010–2014 in Kuwait [50], while in Qatar the prevalence was 51% in children with ARI during 2010–2011 [35], 45.7% during 2012–2017 [51], and 19.7% during 2012–2017 [52]. The prevalence in Qatar was similar to that in this study and to prevalences reported in Saudi Arabia in earlier studies [41]. The prevalence of HOPV in Riyadh is higher than that reported in other cities in Saudi Arabia (6.5–45.3%). This is probably due to the fact that most studies are carried out in Riyadh (14) rather than in other cities (4) in Saudi Arabia [41].

The reported primers (BG10 and F1) were successfully used for amplification of the ectodomain of the G protein gene of group A and group B orthopneumovirus [24]. We investigated the evolutionary dynamics of HOPV with a focus on the ON1 and BA genotypes of HOPV-A and HOPV-B in Riyadh, Saudi Arabia. Current Saudi strains were categorized into the ON1 (with 72 nt duplication) and BA (with 60 nt duplication) groups of the HOPV-A and HOPV-B genotypes, respectively. A new genotyping method for phylogenetic classification was employed, as suggested by Goya and colleagues [12]. This is the first study from the region that used the new method for the classification of ON1 genotypes into different lineages. HOPVA-ON1 genotypes were further categorized into subgenotype GA-2.3 and three different lineages GA-2.3.5, GA-2.3.6a, and GA-2.3.6b. The lineage GA-2.3.5 includes sequences from Argentina, Canada, China, New Zealand, and the USA from 2010 to 2015. Other lineages, GA-2.3.6a and GA-2.3.6b, form a separate group and had sequences from a single country, India (2011 and 2012) or Spain (2014 and 2015), respectively. HOPVA-NA1 genotypes were reported in Riyadh during 2008–2009 [42]. In subsequent studies, the emergence of the HOPVA-ON1 genotype was seen in Riyadh for the first time in 2014 [7] and its dominance was reported in Riyadh in 2016, where there was a significant jump in the prevalence of ON1 (97%) within 15 months, replacing most of the NA1 genotype (3%) [9]. In a subsequent study, the total replacement of NA1 by ON1 was seen in 2017 in Jeddah [44] and during the 2019–2020 winter in Riyadh [8].

Similar ON1 genotype replacements of a previously dominant genotype had been reported in different geographical regions including Thailand, Kenya, and Japan [16,27,52,53]. Past studies have shown that the ON1 genotype has spread rapidly after it was reported in 2010 [52,54]. The replacement of NA1 by ON1 was far more rapid than the replacement of GA2 by GA5, and this can be attributed to a duplication region that possibly gives this genotype a survival advantage over other HOPV-A strains [53,54].

Orthopneumovirus BA genotypes were also classified using the new method [12]. The GB genotypes of HOPV-B were further classified into the GB-5.0 subgenotype and a single lineage, GB-5.0.5a. This single lineage includes sequences from Argentina, England, New Zealand, and the USA for the years 2014 and 2016. These results indicate a mixed orthopneumovirus GA lineage and a single GB lineage circulating during the study period. The circulation of the ON1 and BA genotypes had also been reported earlier around the world. In Northern Thailand the ON1 genotype (49%) of HOPV-A and the BA9 genotype (41%) of HOPV-B were in circulation during 2020–2021 [27]. In Jordan studies revealed the predominance of the HOPV-A ON1 (11.4%) and HOPV-B BA9 (17.1%) genotype strains during 2022 and 2023 [55]. In another study, in Korea in 2019–2022, all HOPV-A strains (56) belonged to the ON1 genotype, whereas all HOPV-B strains (77) belonged to the BA genotype [56]. This study further validates the fact that a decrease in diversity has been observed among HOPV genotypes, with the ON1 and BA genotypes becoming the sole lineages over the last two decades [57].

The HOPV-B G gene seems to be under higher selection pressure as analysis of mutations at the ectodomain revealed more mutations in the HOPV-B G gene than in the HOPV-A G gene. Both genotypes showed a higher rate of mutations at the second hypervariable region of the G gene compared to its analogous region. These duplicated regions accumulate mutations with time that contribute towards additional variability in the genotype. The ON1 and BA strains have shown a progressive accumulation of mutations in the second hypervariable region. In 2014, only two mutations were reported in the duplicated ON1 region [7] of HOPV in Riyadh compared to the prototype strain from Canada. More mutations (11) were reported in the region in a subsequent study in 2016 [9]. Mutations in the region accumulated further during the 2019–2020 winter, as reported in 2023 [8]. A similar progressive accumulation of mutations was also reported in the duplicated region of the HOPVB-BA genotype in subsequent studies from the region [7,9]. The accumulation of mutations indicated higher selection pressure in the second hypervariable region of the HOPV-A and HOPV-B G genes. Higher entropy values at some amino acid positions, like ON1 (L298P/S/T) and BA (P216S/L) in the G protein, also indicate higher selection pressures within these genotypes. This again confirms the high evolutionary pressure in this region of the G protein. L274P substitution is seen in the present investigation and was also reported earlier [7,8,9,58,59]. This substitution has been associated with a loss of glycosylation in some studies [60]. It has also been associated with resistance to neutralizing antibody activity [61,62,63] and antibody escape mutants [64,65]. The substitutions Y280H and Y304H are located within known antigenic sites of the G protein in the analogous and duplicate regions, respectively, and have also been reported previously [66]. Studies have shown a progressive increase in the accumulation of these substitutions with time from the Riyadh region [7,8,9]. In 2014, no Y280H substitution was seen in the analogous region while half of the sequences had a Y304H substitution in the duplication region [7]. In another study, in 2016, Y280H substitution was seen in a single sample while Y304 substitution was seen in 17 of the 32 samples [9]. Similarly, a study in 2022 reported a higher number of Y280H substitutions (4) and the highest number of Y304H substitutions (23) among its 37 samples [8]. From these findings it can be speculated that the Y-to-H substitution at amino acid position 304 in the duplicated region is incorporated first, followed by a substitution at the 280 site in the analogous region.

The glycosylation of amino acids affects the antigenicity of the viral proteins. The presence of potential N- and O-linked glycosylation sites was observed in both the ON1 and BA genotypes. Four N-glycosylation sites in the ON1 genotype were identified outside the analogous and duplicate regions in all ON1 study sequences and a single O-linked glycosylation site was observed in the majority of ON1 sequences, and this was also reported earlier [7,8,9,18]. In the BA genotype, four N-glycosylation sites were observed outside the analogous and duplicate regions and a single site (at the 258 amino acid position) was observed in a majority of the samples at the juncture of the analogous and duplicate regions. The N-glycosylation site within the duplicated region was observed in one of the samples from Riyadh reported earlier [7]. Thus, a progressive accumulation of N-glycosylation sites within the analogous and duplicate regions in BA genotypes is evident. The higher glycosylation frequency in this region further contributes towards its antigenic variability, which may further support immune evasion.

Although the samples analyzed in the present study were from a single epidemic season, we showed the circulation in Saudi Arabia of two different genotypes of HOPV-A and HOPV-B with duplication in the G protein gene. We included the ectodomain of the G protein gene for analysis as suggested [12]. These genetic and antigenic variations in the neutralizing agent, like the G protein gene, provide an opportunity to access the extent of variability in the gene. This may be of assistance in the design of a vaccine against HOPV that targets the G protein since F protein-based vaccines only provide protection in a limited group of individuals.

## Figures and Tables

**Table 1:** Clinical manifestations of the study patients.

Age Group (Months)	No. of Clinical Cases	Gender		Symptoms (%)				RSV	
		Males	Females	Fever	Cough	Nasal Discharge	Sore Throat	+Ve	−Ve
1–6	290	167	123	283	281	284	273	45	245
7–12	205	123	82	204	200	189	192	31	174
13–24	145	93	52	133	131	132	136	22	123
**Total**	**640**	**383**	**257**	**620**	**612**	**605**	**601**	**98**	**542**

## Data Availability

The original contributions presented in this study are included in the Supplementary Material. Further inquiries can be directed to the corresponding author.

## Supplementary Materials

The following supporting information can be downloaded at https://www.mdpi.com/article/10.3390/cimb47100826/s1.
