# Supplementary material for: Equal Prevalence of Genotypes ON1 and BA of Human Orthopneumovirus in Riyadh, Saudi Arabia, in 2022
Source: Curr Issues Mol Biol. 2025 Oct 8;47(10):826. doi: 10.3390/cimb47100826 (PMC12564530; doi:10.3390/cimb47100826)
Supplement: Supplementary file 1 [file cimb-47-00826-s001.zip › cimb-3856887-supplementary-2-1(1).pdf]

Table S1. List of HOPV-A ON1 strains used in the study

S.No.	Accession No	Genotype/Sub-genotype	Country	Year of collection
1.	AY911262	GA-1	USA	1956
2.	JX198138	GA-1	USA	1961
3.	KJ723474	GA-1	USA	1989
4.	KU316165	GA-1	USA	1987
5.	Z33427	GA-1	Uruguay	1990
6.	MG642063	GA-2	USA	1982
7.	HQ731716	GA-2	England	1981
8.	KJ723483	GA-2.1	USA	1984
9.	KP258723	GA-2.1	USA	1986
10.	KU316098	GA-2.1	USA	1984
11.	MG642070	GA-2.1	USA	1986
12.	JF920062	GA-2.2	USA	1998
13.	JX069801	GA-2.2	USA	1998
14.	KJ723492	GA-2.2	USA	1990
15.	KP258700	GA-2.2	USA	1985
16.	KP258743	GA-2.2	USA	1998
17.	KU316092	GA-2.2	USA	1991
18.	MG642030	GA-2.2	USA	1988
19.	JX069798	GA-2.3.0	USA	2001
20.	KP119748	GA-2.3.0	Hong Kong	2012
21.	KU316118	GA-2.3.0	USA	1996
22.	KU950573	GA-2.3.0	USA	2006
23.	MG642033	GA-2.3.0	USA	1994
24.	JQ901452	GA-2.3.1	Netherlands	2001
25.	JX015480	GA-2.3.1	Netherlands	2007
26.	JX015486	GA-2.3.2b	Netherlands	2005
27.	KJ627284	GA-2.3.2b	Peru	2010
28.	KJ627336	GA-2.3.2b	Peru	2008
29.	KJ627256	GA-2.3.3	Peru	2009
30.	KJ627349	GA-2.3.3	Peru	2009
31.	KP317953	GA-2.3.3	Kenya	2012
32.	KY654511	GA-2.3.3	Philippines	2013
33.	KC731483	GA-2.3.4	India	2011
34.	KY460517	GA-2.3.4	Taiwan	2010
35.	KY654508	GA-2.3.4	Philippines	2012
36.	MF001051	GA-2.3.4	USA	2015
37.	MF001053	GA-2.3.4	USA	2015
38.	JN257693	GA-2.3.5	Canada	2010
39.	KJ672470	GA-2.3.5	USA	2012
40.	KT285064	GA-2.3.5	China	2014
41.	KU950540	GA-2.3.5	USA	2013
42.	KU950531	GA-2.3.5	USA	2013
43.	KU950550	GA-2.3.5	USA	2012

Table S1. List of HOPV-A ON1 strains used in the study

44.	KU950556	GA-2.3.5	USA	2012
45.	KU950560	GA-2.3.5	USA	2013
46.	KU950651	GA-2.3.5	USA	2012
47.	KU950670	GA-2.3.5	USA	2012
48.	KX765917	GA-2.3.5	New Zealand	2013
49.	KX765932	GA-2.3.5	New Zealand	2015
50.	KX765941	GA-2.3.5	New Zealand	2015
51.	KX765971	GA-2.3.5	New Zealand	2013
52.	KX894807	GA-2.3.5	USA	2013
53.	KY883567	GA-2.3.5	Argentina	2015
54.	KT326808	GA-2.3.6a	Spain	2015
55.	KT326810	GA-2.3.6a	Spain	2014
56.	KF246638	GA-2.3.6b	India	2012
57.	KF246641	GA-2.3.6b	India	2012
58.	KF246640	GA-2.3.6b	India	2012
59.	KC731482	GA-2.3.6b	India	2011
60.	HQ731715	GA-3.0.0	England	1976
61.	KP258715	GA-3.0.0	USA	1988
62.	KU316137	GA-3.0.0	USA	1979
63.	KU316149	GA-3.0.0	USA	1977
64.	KP258699	GA-3.0.1	USA	1985
65.	KU316133	GA-3.0.1	USA	1990
66.	MG642031	GA-3.0.1	USA	1982
67.	KJ723465	GA-3.0.2	USA	1992
68.	KP258701	GA-3.0.2	USA	1994
69.	KU316104	GA-3.0.2	USA	1991
70.	KU316161	GA-3.0.2	USA	1993
71.	KU316170	GA-3.0.2	USA	1995
72.	JQ901455	GA-3.0.3a	Netherlands	2002
73.	JX069802	GA-3.0.3a	USA	1998
74.	KM360090	GA-3.0.3a	USA	2001
75.	KY967364	GA-3.0.3a	USA	2015
76.	JX513302	GA-3.0.3b	Brazil	2002
77.	JX513334	GA-3.0.3b	Brazil	2003
78.	KF826826	GA-3.0.4b	Mexico	2004
79.	KF826827	GA-3.0.4b	Argentina	2004
80.	KF826850	GA-3.0.4b	USA	2008
81.	KF973333	GA-3.0.4b	USA	2002
82.	KC297367	GA-3.0.5b	China	2012
83.	KF826832	GA-3.0.5b	Italy	2009
84.	KX765933	GA-3.0.5b	New Zealand	2012
85.	MF001038	GA-3.0.5b	USA	2015

Table S2. List of HOPV-B BA strains used in the study

S.NO	Accession No	Genotype/Sub-genotype	Country	Year of collection
1.	AY352550	GB-1	USA	1977
2.	HQ731711	GB-1	England	1965
3.	JX198143	GB-1	USA	1962
4.	AF013254	GB-2	USA	1985
5.	HQ731708	GB-2	England	1985
6.	HQ731718	GB-2	England	1986
7.	JX198165	GB-2	USA	1993
8.	KP258712	GB-2	USA	1979
9.	KU316127	GB-2	USA	1991
10.	KU316173	GB-2	USA	1984
11.	KU316175	GB-2	USA	1985
12.	KU316181	GB-2	USA	1990
13.	KU316182	GB-2	USA	1990
14.	M73540	GB-2	USA	1980
15.	MG642036	GB-2	USA	1989
16.	MG642043	GB-2	USA	1982
17.	JX198147	GB-3	USA	1993
18.	AY333361	GB-3	Uruguay	1990
19.	JX198166	GB-3	USA	1993
20.	AF193331	GB-4	South Korea	1991
21.	HQ731722	GB-4	England	1994
22.	JF704214	GB-4	South Africa	1997
23.	JX198160	GB-4	USA	1993
24.	MG642062	GB-4	USA	1996
25.	KF826853	GB-5.0.0	Germany	2008
26.	KP258713	GB-5.0.0	USA	1993
27.	KP317923	GB-5.0.0	Kenya	2012
28.	KU316134	GB-5.0.0	USA	1994
29.	DQ227364	GB-5.0.1	Argentina	1999
30.	JX576761	GB-5.0.1	Netherlands	2002
31.	JX576762	GB-5.0.1	Netherlands	2002
32.	KF826829	GB-5.0.1	Mexico	2005
33.	KJ939919	GB-5.0.1	Vietnam	2009
34.	MF185752	GB-5.0.1	USA	2002
35.	AY333364	GB-5.0.1	Argentina	2003
36.	HQ731688	GB-5.0.2	Scotland	2006
37.	JX576742	GB-5.0.2	Netherlands	2009
38.	KC297492	GB-5.0.2	China	2009
39.	KF826845	GB-5.0.2	Argentina	2008
40.	KF826843	GB-5.0.2	Mexico	2008
41.	KJ627302	GB-5.0.2	Peru	2008
42.	KJ939926	GB-5.0.2	Vietnam	2010
43.	KU950484	GB-5.0.2	USA	2013
44.	KU950619	GB-5.0.2	USA	2012
45.	KX765943	GB-5.0.2	New Zealand	2013

Table S2. List of HOPV-B BA strains used in the study

46.	KY249662	GB-5.0.2	England	2013
47.	JX704224	GB-5.0.3	South Africa	2007
48.	JN032117	GB-5.0.3	USA	2006
49.	KC476394	GB-5.0.3	Cuba	2009
50.	KF246627	GB-5.0.3	India	2010
51.	KF826839	GB-5.0.3	Argentina	2006
52.	MG431252	GB-5.0.3	Brazil	2010
53.	KJ939929	GB-5.0.4a	Vietnam	2010
54.	KJ939932	GB-5.0.4a	Vietnam	2010
55.	KM402687	GB-5.0.4a	Spain	2013
56.	KU950467	GB-5.0.4a	USA	2014
57.	KX655648	GB-5.0.4a	Jordan	2013
58.	KX765912	GB-5.0.4a	New Zealand	2014
59.	KX765957	GB-5.0.4a	New Zealand	2014
60.	KX765962	GB-5.0.4a	New Zealand	2013
61.	KY249657	GB-5.0.4a	England	2014
62.	KY249670	GB-5.0.4a	England	2012
63.	KY249677	GB-5.0.4a	England	2012
64.	KY883571	GB-5.0.4a	Argentina	2015
65.	JN032115	GB-5.0.4b	USA	2006
66.	JX576746	GB-5.0.4b	Netherlands	
67.	JX576730	GB-5.0.4b	Belgium	
68.	JX576751	GB-5.0.4b	Netherlands	2008
69.	JX976389	GB-5.0.4b	Cuba	2011
70.	KF300963	GB-5.0.4b	Panama	2011
71.	KF826860	GB-5.0.4b	Italy	2009
72.	KJ627285	GB-5.0.4b	Peru	2011
73.	JX489429	GB-5.0.4c	Brazil	2011
74.	JX976333	GB-5.0.4c	Cuba	2010
75.	JX976378	GB-5.0.4c	Cuba	2011
76.	KJ627262	GB-5.0.4c	Peru	2009
77.	KJ939928	GB-5.0.4c	Vietnam	2010
78.	KM402730	GB-5.0.4c	Spain	2014
79.	KP317928	GB-5.0.4c	Kenya	2011
80.	KU950477	GB-5.0.4c	USA	2014
81.	KU950588	GB-5.0.4c	USA	2014
82.	KX655649	GB-5.0.4c	Jordan	2013
83.	KX655654	GB-5.0.4c	Jordan	2011
84.	KX765949	GB-5.0.4c	New Zealand	2011
85.	KY249658	GB-5.0.4c	England	2013
86.	KX765906	GB-5.0.5a	New Zealand	2015
87.	KY249683	GB-5.0.5a	England	2016
88.	KY684758	GB-5.0.5a	USA	2016
89.	MG773268	GB-5.0.5a	Argentina	2016
90.	MG839547	GB-5.0.5a	Argentina	2016
91.	JF704213	GB-6	South Africa	1998

Table S2. List of HOPV-B BA strains used in the study

92.	MF185751	GB-6	USA	2002
93.	KC297428	GB-7	China	2009
94.	KC297462	GB-7	China	2010
95.	KC297466	GB-7	China	2011
96.	KF246637	GB-7	India	2012
97.	KT781406	GB-7	China	2014

Table S3: List of HOPV-A and HOPV-B positive clinical samples with their genotypes, subgenotypes, lineages and corresponding GenBank accession numbers.

**HOPVA**					
**S.No.**	**Clinical sample**	**Genotype**	**GeneBank Accession No.**	**Subgenotype**	**Lineage**
1	F-6/RSV-A/Saudi-Arabia/2022	ON1	PQ798904	GA-2.3	GA-2.3.5
2	F-8/RSV-A/Saudi-Arabia/2022	ON1	PQ798905	GA-2.3	GA-2.3.5
3	F-9/RSV-A/Saudi-Arabia/2022	ON1	PQ798906	GA-2.3	GA-2.3.5
4	F-39/RSV-A/Saudi-Arabia/2022	ON1	PQ798907	GA-2.3	GA-2.3.6a
5	F-66/RSV-A/Saudi-Arabia/2022	ON1	PQ798908	GA-2.3	GA-2.3.6a
6	F-77/RSV-A/Saudi-Arabia/2022	ON1	PQ798909	GA-2.3	GA-2.3.6a
7	F-78/RSV-A/Saudi-Arabia/2022	ON1	PQ798910	GA-2.3	GA-2.3.6a
8	F-84/RSV-A/Saudi-Arabia/2022	ON1	PQ798911	GA-2.3	GA-2.3.6a
9	F-87/RSV-A/Saudi-Arabia/2022	ON1	PQ798912	GA-2.3	GA-2.3.5
10	F-88/RSV-A/Saudi-Arabia/2022	ON1	PQ798913	GA-2.3	GA-2.3.6a
11	BG-17/RSV-A/Saudi-Arabia/2022	ON1	PQ798914	GA-2.3	GA-2.3.6a
12	BG-56/RSV-A/Saudi-Arabia/2022	ON1	PQ798915	GA-2.3	GA-2.3.6b
13	BG-61/RSV-A/Saudi-Arabia/2022	ON1	PQ798916	GA-2.3	GA-2.3.6a
14	BG-71/RSV-A/Saudi-Arabia/2022	ON1	PQ798917	GA-2.3	GA-2.3.6a
15	BG-74/RSV-A/Saudi-Arabia/2022	ON1	PQ798918	GA-2.3	GA-2.3.6a
16	BG-90/RSV-A/Saudi-Arabia/2022	ON1	PQ798919	GA-2.3	GA-2.3.6a
17	BG-10/RSV-A/Saudi-Arabia/2022	ON1	PQ798920	GA-2.3	GA-2.3.6b
18	BG-11/RSV-A/Saudi-Arabia/2022	ON1	PQ798921	GA-2.3	GA-2.3.6a
19	BG-33/RSV-A/Saudi-Arabia/2022	ON1	PQ798922	GA-2.3	GA-2.3.6b
20	BG-38/RSV-A/Saudi-Arabia/2022	ON1	PQ798923	GA-2.3	GA-2.3.6a
21	BG-59/RSV-A/Saudi-Arabia/2022	ON1	PQ798924	GA-2.3	GA-2.3.5
22	BG-60/RSV-A/Saudi-Arabia/2022	ON1	PQ798925	GA-2.3	GA-2.3.6a
23	BG-83/RSV-A/Saudi-Arabia/2022	ON1	PQ798926	GA-2.3	GA-2.3.6a
24	BG-99/RSV-A/Saudi-Arabia/2022	ON1	PQ798927	GA-2.3	GA-2.3.6b
25	BG-54/RSV-A/Saudi-Arabia/2022	ON1	PQ798928	GA-2.3	GA-2.3.6b
26	F-29/RSV-A/Saudi-Arabia/2022	ON1	PQ798929	GA-2.3	GA-2.3.6a
27	F-1/RSV-A/Saudi-Arabia/2022	ON1	PQ798930	GA-2.3	GA-2.3.6a
28	F-5/RSV-A/Saudi-Arabia/2022	ON1	PQ798931	GA-2.3	GA-2.3.6a
29	F-20/RSV-A/Saudi-Arabia/2022	ON1	PQ798932	GA-2.3	GA-2.3.5
30	F-32/RSV-A/Saudi-Arabia/2022	ON1	PQ798933	GA-2.3	GA-2.3.6a
31	BG-94/RSV-A/Saudi-Arabia/2022	ON1	PQ798934	GA-2.3	GA-2.3.6a
32	BG-15/RSV-A/Saudi-Arabia/2022	ON1	PQ798935	GA-2.3	GA-2.3.6a
33	BG-47/RSV-A/Saudi-Arabia/2022	ON1	PQ798936	GA-2.3	GA-2.3.6b
**HOPVB**					
**S.No.**	**Clinical sample**	**Genotype**	**GeneBank Accession No.**	Subgenotype	Lineage
1	BG-2/RSV-B/Saudi-Arabia/2022	BA	PQ798831	GB-5.0	GB-5.0.5a

Table S3: List of HOPV-A and HOPV-B positive clinical samples with their genotypes, subgenotypes, lineages and corresponding GenBank accession numbers.

2	BG-3/RSV-B/Saudi-Arabia/2022	BA	PQ798832	GB-5.0	GB-5.0.5a
3	BG-4/RSV-B/Saudi-Arabia/2022	BA	PQ798833	GB-5.0	GB-5.0.5a
4	BG-13/RSV-B/Saudi-Arabia/2022	BA	PQ798834	GB-5.0	GB-5.0.5a
5	BG-14/RSV-B/Saudi-Arabia/2022	BA	PQ798835	GB-5.0	GB-5.0.5a
6	BG-18/RSV-B/Saudi-Arabia/2022	BA	PQ798836	GB-5.0	GB-5.0.5a
7	BG-19/RSV-B/Saudi-Arabia/2022	BA	PQ798837	GB-5.0	GB-5.0.5a
8	BG-21/RSV-B/Saudi-Arabia/2022	BA	PQ798838	GB-5.0	GB-5.0.5a
9	BG-26/RSV-B/Saudi-Arabia/2022	BA	PQ798839	GB-5.0	GB-5.0.5a
10	BG-27/RSV-B/Saudi-Arabia/2022	BA	PQ798840	GB-5.0	GB-5.0.5a
11	BG-34/RSV-B/Saudi-Arabia/2022	BA	PQ798841	GB-5.0	GB-5.0.5a
12	BG-52/RSV-B/Saudi-Arabia/2022	BA	PQ798842	GB-5.0	GB-5.0.5a
13	BG-64/RSV-B/Saudi-Arabia/2022	BA	PQ798843	GB-5.0	GB-5.0.5a
14	BG-75/RSV-B/Saudi-Arabia/2022	BA	PQ798844	GB-5.0	GB-5.0.5a
15	BG-76/RSV-B/Saudi-Arabia/2022	BA	PQ798845	GB-5.0	GB-5.0.5a
16	BG-93/RSV-B/Saudi-Arabia/2022	BA	PQ798846	GB-5.0	GB-5.0.5a
17	BG-96/RSV-B/Saudi-Arabia/2022	BA	PQ798847	GB-5.0	GB-5.0.5a
18	BG-101/RSV-B/Saudi-Arabia/2022	BA	PQ798848	GB-5.0	GB-5.0.5a
19	BG-102/RSV-B/Saudi-Arabia/2022	BA	PQ798849	GB-5.0	GB-5.0.5a
20	BG-108/RSV-B/Saudi-Arabia/2022	BA	PQ798850	GB-5.0	GB-5.0.5a
21	BG-110/RSV-B/Saudi-Arabia/2022	BA	PQ798851	GB-5.0	GB-5.0.5a
22	F-16/RSV-B/Saudi-Arabia/2022	BA	PQ798852	GB-5.0	GB-5.0.5a
23	F-28/RSV-B/Saudi-Arabia/2022	BA	PQ798853	GB-5.0	GB-5.0.5a
24	F-30/RSV-B/Saudi-Arabia/2022	BA	PQ798854	GB-5.0	GB-5.0.5a
25	F-42/RSV-B/Saudi-Arabia/2022	BA	PQ798855	GB-5.0	GB-5.0.5a
26	F-43/RSV-B/Saudi-Arabia/2022	BA	PQ798856	GB-5.0	GB-5.0.5a
27	F-50/RSV-B/Saudi-Arabia/2022	BA	PQ798857	GB-5.0	GB-5.0.5a
28	F-49/RSV-B/Saudi-Arabia/2022	BA	PQ798858	GB-5.0	GB-5.0.5a
29	F-57/RSV-B/Saudi-Arabia/2022	BA	PQ798859	GB-5.0	GB-5.0.5a
30	F-68/RSV-B/Saudi-Arabia/2022	BA	PQ798860	GB-5.0	GB-5.0.5a
31	F-70/RSV-B/Saudi-Arabia/2022	BA	PQ798861	GB-5.0	GB-5.0.5a
32	F-72/RSV-B/Saudi-Arabia/2022	BA	PQ798862	GB-5.0	GB-5.0.5a
33	F-80/RSV-B/Saudi-Arabia/2022	BA	PQ798863	GB-5.0	GB-5.0.5a
34	BG-7/RSV-B/Saudi-Arabia/2022	BA	PQ798864	GB-5.0	GB-5.0.5a
35	BG-31/RSV-B/Saudi-Arabia/2022	BA	PQ798865	GB-5.0	GB-5.0.5a
36	BG-44/RSV-B/Saudi-Arabia/2022	BA	PQ798866	GB-5.0	GB-5.0.5a
37	BG-43/RSV-B/Saudi-Arabia/2022	BA	PQ798867	GB-5.0	GB-5.0.5a
38	BG-45/RSV-B/Saudi-Arabia/2022	BA	PQ798868	GB-5.0	GB-5.0.5a
39	BG-46/RSV-B/Saudi-Arabia/2022	BA	PQ798869	GB-5.0	GB-5.0.5a
40	BG-48/RSV-B/Saudi-Arabia/2022	BA	PQ798870	GB-5.0	GB-5.0.5a
41	BG-49/RSV-B/Saudi-Arabia/2022	BA	PQ798871	GB-5.0	GB-5.0.5a
42	BG-50/RSV-B/Saudi-Arabia/2022	BA	PQ798872	GB-5.0	GB-5.0.5a

Table S3: List of HOPV-A and HOPV-B positive clinical samples with their genotypes, subgenotypes, lineages and corresponding GenBank accession numbers.

43	BG-51/RSV-B/Saudi-Arabia/2022	BA	PQ798873	GB-5.0	GB-5.0.5a
44	BG-53/RSV-B/Saudi-Arabia/2022	BA	PQ798874	GB-5.0	GB-5.0.5a
45	BG-65/RSV-B/Saudi-Arabia/2022	BA	PQ798875	GB-5.0	GB-5.0.5a
46	BG-72/RSV-B/Saudi-Arabia/2022	BA	PQ798876	GB-5.0	GB-5.0.5a
47	BG-109/RSV-B/Saudi-Arabia/2022	BA	PQ798877	GB-5.0	GB-5.0.5a

Table S6: Positive selected sites on HOPV-A ON1 genotypes in terms of selection pressure

Residue Number	MEME (p-Value)	FUBAR [p (α < β)]
72	√	X
73	√	√
80	X	√
81	X	√
82	√	X
178	√	X
217	√	√
223	√	X
238	√	√
272	√	√
293	√	X
298	√	√
312	X	√

Table S7: Positive selected sites on HOPV-B BA genotype in terms of selection pressure

Residue Number	MEME (p-Value)	FUBAR [p (α < β)]
71	√	✕
73	√	✕
74	√	✓
79	√	✕
81	√	✕
83	√	✕
89	√	✕
92	√	✕
99	√	✕
110	√	✕
113	√	✕
126	√	✕
128	√	✕
150	√	✕
168	√	✕
177	√	✕
222	√	✕
231	√	✓
234	√	✕
244	√	✕
248	√	✕
260	√	✓
266	√	✕
293	√	✓
298	√	✕
